# Other PHAC publications

**DOI:** 10.24095/hpcdp.44.5.07

**Published:** 2024-05

**Researchers from the Public Health Agency of Canada also contribute to work published in other journals and books. Look for the following articles published in 2024:**

Hassen N, Lacaille D, Xu A, […], **Lang JJ**, et al. National burden of rheumatoid arthritis in Canada, 1990–2019: findings from the Global Burden of Disease Study 2019 – a GBD collaborator-led study. RMD Open. 2024;10:e003533. https://doi.org/10.1136/rmdopen-2023-003533

**Idzerda L, Corrin T, Lazarescu C, Couture A, Vallires E, Khan S, […], Garcia AJ**. Public policy interventions to mitigate household food insecurity in Canada: a systematic review. Public Health Nutr. 2024;27(1):e83. https://doi.org/10.1017/S1368980024000120

**Pagaduan JE, Lazarescu C, Vallieres E, […], Zuckermann AME, Idzerda L**. The impacts of the Nutrition North Canada program on the accessibility and affordability of perishable, nutritious foods among eligible communities: a scoping review. Int J Circumpolar Health. 2024;83(1):2313255. https://doi.org/10.1080/22423982.2024.2313255

**Prince SA**, Dempsey PC, Reed JL, […], **Merucci K, Lang JJ**. The effect of sedentary hehaviour on cardiorespiratory fitness: a systematic review and meta-analysis. Sports Med. 2024. https://doi.org/10.1007/s40279-023-01986-y

**Prince SA, Lang JJ, Betancourt M, Toigo S, Roberts KC**. Sedentary time at school and work in Canada. Can J Public Health. 2024. https://doi.org/10.17269/s41997-023-00835-9

